# The cost-effectiveness of different types of educational interventions in type II diabetes mellitus: A systematic review

**DOI:** 10.3389/fphar.2022.953341

**Published:** 2022-07-22

**Authors:** Wan Nur Liyana Hazwani Wan Rohimi, Nurul Ain Mohd Tahir

**Affiliations:** Faculty of Pharmacy, Universiti Kebangsaan Malaysia, Kuala Lumpur, Malaysia

**Keywords:** pharmacoeconomic, systematic review, diabetes mellitus, educational intervention, cost-effectiveness

## Abstract

**Aims:** Educational interventions are effective to improve peoples’ self-efficacy in managing diabetes complications and lifestyle changes. This systematic review aims to assess and compare various aspects of educational interventions and to provide updated pharmacoeconomics data.

**Methods:** Literature searches were conducted using databases such as EBSCOhost, Ovid, PubMed, Scopus, and Web of Science. Outcomes such as study characteristics, costs, medication adherence, effectiveness and were narratively summarized, and the quality of each article was assessed.

**Results:** A total of 27 studies were retrieved. The types of educational interventions were classified as face-to-face strategy, structured programs, telemedicine health education, a combination approach, and others. All types of educational interventions (N = 24, 89%) were reported to be cost-effective. The cost-effectiveness of the other two studies was considered to be not cost-effective while the outcome of one study could not be determined. The majority of the studies (N = 24, 89%) had moderate-quality evidence whereas thirteen (48%) studies were regarded to provide high-quality economic evaluations.

**Conclusion:** All types of educational interventions are highly likely to be cost-effective. The quality of economic evaluations is moderate but the most cost-effective types of educational interventions could not be determined due to variations in the reporting and methodological conduct of the study. A high-quality approach, preferably utilizing the societal perspective over a long period, should be standardized to conduct economic evaluations for educational interventions in T2DM.

**Systematic Review Registration:** website, identifier registration number.

## Introduction

Diabetes affects 422 million people globally, with the majority living in low-and middle-income countries (WHO, 2021). This number has significantly increased as 170 million people were diagnosed in the 2000s, and currently, it has exceeded the projected number of 366 million people having diabetes by 2030 (Wild et al., 2004). Out of these, type II diabetes mellitus (T2DM) usually accounted for nine out of every ten people (Zimmet, 2003). T2DM is commonly associated with obesity and other comorbidities, including hypertension, cardiovascular-related diseases, and microvascular disorders that could lead to complications that may impact people’s quality of life (Keith Campbell, 2009). A study in South Australia reported that 56.6% of cases of mortality of T2DM people were caused by cardiovascular-related diseases, followed by 15.0 and 10.9% due to unnatural deaths and infections, respectively (Zhou and Byard, 2018). The economic burden of diabetes mellitus is equally substantial. International Diabetes Federation (IDF) reported enormous expenses of US$850 billion for the management of diabetes mellitus globally in 2017, and it was projected to increase up to US$958 billion by 2045 (Cho et al., 2018). In the United States, an estimated cost of $327 billion was for diabetes, accounting for 25% of all healthcare spending (ADA, 2018). The burden of expenses is amplified in T2DM people requiring an additional cost of US$9,643 as compared to non-diabetes people and the difference can increase up to US$18,057 after 8 years of diagnosis (Zimmet, 2003). The high cost of diabetes care is a significant problem in today’s healthcare system as well as a burden on people.

Educational interventions are useful and successful strategies that can contribute to positive outcomes in peoples’ knowledge and attitude in managing their T2DM as well as effectively improving people’s glycaemic control (Ahmed et al., 2015). The effectiveness of these interventions which includes various forms and components in preventing the onset and delaying the progression of complications of T2DM have always been widely studied and reported in an abundant number of studies (Merakou et al., 2015; Zhang and Chu, 2018; Mohamed et al., 2019). Various beneficial methods of delivering educational interventions had been accomplished in improving diabetes self-care, however, these studies revealed inconsistency in the determination of the elements of educational interventions and thus became the major barrier to understanding the outcomes of educational research in diabetes (Sigurdardottir et al., 2007). Furthermore, a diversity of educational programs did not yield consistent results on measures of metabolic control (Loveman et al., 2003). For example, the diversity of theories concerning delivery, teaching methods, content, and depth of educational interventions may have an impact on the HbA1c and metabolic outcomes of T2DM patients (Loveman et al., 2003; Sigurdardottir et al., 2007).

Similarly, numerous systematic reviews of the cost-effectiveness of educational interventions have resulted in equivocal conclusions (Loveman et al., 2003; Odnoletkova et al., 2014). Furthermore, it ignores the variety of educational intervention aspects and economic evaluation methodological conducts that could influence the cost-effectiveness decision. This evidence could potentially fill in the existing gaps and provide updated pharmacoeconomics data for the healthcare policymakers in determining which elements of educational interventions are effective and economically practical to be implemented in the provision of health care for T2DM people.

## Methods

Preferred Reporting Items for Systematic Reviews and Meta-Analyses (PRISMA) was used as a guide in reporting the economic evaluation outcomes in this systematic review (Moher et al., 2009).Search strategy

Major databases selected in generating the search included EBSCOhost, Ovid, PubMed, Scopus, and Web of Science. The main search terms such as “cost-effectiveness”, “educational interventions”, “medication adherence”, and “type II diabetes mellitus” were used. The medical subject heading (MeSH) was employed where applicable. Subsequent keywords were added in conjunction to the main keywords by connecting them using Boolean Operator “OR” to broaden the search. The subsequent keywords were as follows (“costs” OR “cost analysis” OR “cost utility” OR “cost benefit” OR “health economics” OR “economic evaluation” OR “cost minimi?ation”) (“educational strategies” OR “patient education”) (“medication compliance” OR “medication nonadherence” OR “medication noncompliance” OR “persistence” OR “constancy”) (“diabetes mellitus type II” OR “metabolic disease” OR “non-insulin dependent diabetes” OR “T2DM” OR “NIDDM”). Boolean Operator “AND” was used to connect all the keywords to narrow down and retrieve all the relevant studies. The language of all the literature selected was limited to English. The database was searched for all articles published between the start of the database and the last search date of 28 October 2020.

### Inclusion and exclusion criteria

The eligibility criteria for inclusion of the article include: 1) the study population is those diagnosed with T2DM, 2) The intervention is an educational intervention defined as the number of approaches aim to improve disease knowledge and management skills 3) the comparator is the usual care or any control group specified in the study, 4) the main outcome is the comparative cost-effectiveness value. The primary outcomes mainly focused on the cost-effectiveness of the educational interventions meanwhile the secondary outcomes included the costs, medication adherence, and health effects of the interventions such as HbA1c level and T2DM complications. Only original articles will be included in the review. Articles with study design such as study protocol, pilot study, case reports, editorial, comments, and notes were excluded from the review.

### Study selection

All the article generated was entered into a reference management software (EndNote) to be screened. Duplicates of the article were excluded beforehand. The remaining articles were screened against the inclusion and exclusion criteria based on the titles, followed by the abstracts to obtain the qualified articles. The articles that were relevant to the research topics and fulfilled all the eligibility criteria were selected to be read in full text. The articles that did not meet the criteria were removed and the remaining articles were included in the systematic review. The whole study selection process was conducted by the first reviewer (LH) and further checked by the second reviewer (NAMT) to ensure that an appropriate assessment for inclusion was done.

### Data extraction and synthesis of relevant information

Characteristics of the studies including the type of economic evaluation, study design, the intervention, comparator, perspective, year and type of costs, sources of data, time horizon, and main economic outcomes were extracted.

The information related to the primary and secondary outcomes of the study such as the prevalence of medication adherence, the effectiveness measured by clinical measures or health effects, costs, the outcomes measures, and the cost-effectiveness ratio were further extracted. The costs, medication adherence, and the health effects of the educational interventions were considered the secondary outcomes of the study. The data extracted were analyzed, grouped, and summarized narratively by the first reviewer (LH). The second reviewer (NAMT) was in charge of making sure that the data entered was relevant to the interest of the study.

### Quality assessment

The methodological quality of the retrieved studies was assessed against ten items indicated in the ten-point Drummond checklist (Drummond et al., 2015). The checklist concisely formulated all the essential aspects needed for the judgment of the studies such as follows: the research question, the study description, the effectiveness of the intervention, the identification of health effects and its measurements, the valuation and effects of the interventions, discounting, incremental costs analysis, the determination of uncertainty by sensitivity analysis and discussion relevant to the available health policies. Each item in the checklist was scaled from a score of 1–10, where a score ranging from 1 to 3 indicates poor quality, followed by a score ranging from 4 to 7 reflects a modest-quality while a high-quality score of 8–10 (Doran, 2008). The quality assessment was performed by the first reviewer (LH) and the second reviewer (NAMT) will further validate the quality of the studies.

## Results

### Study selection

**FIGURE 1:**
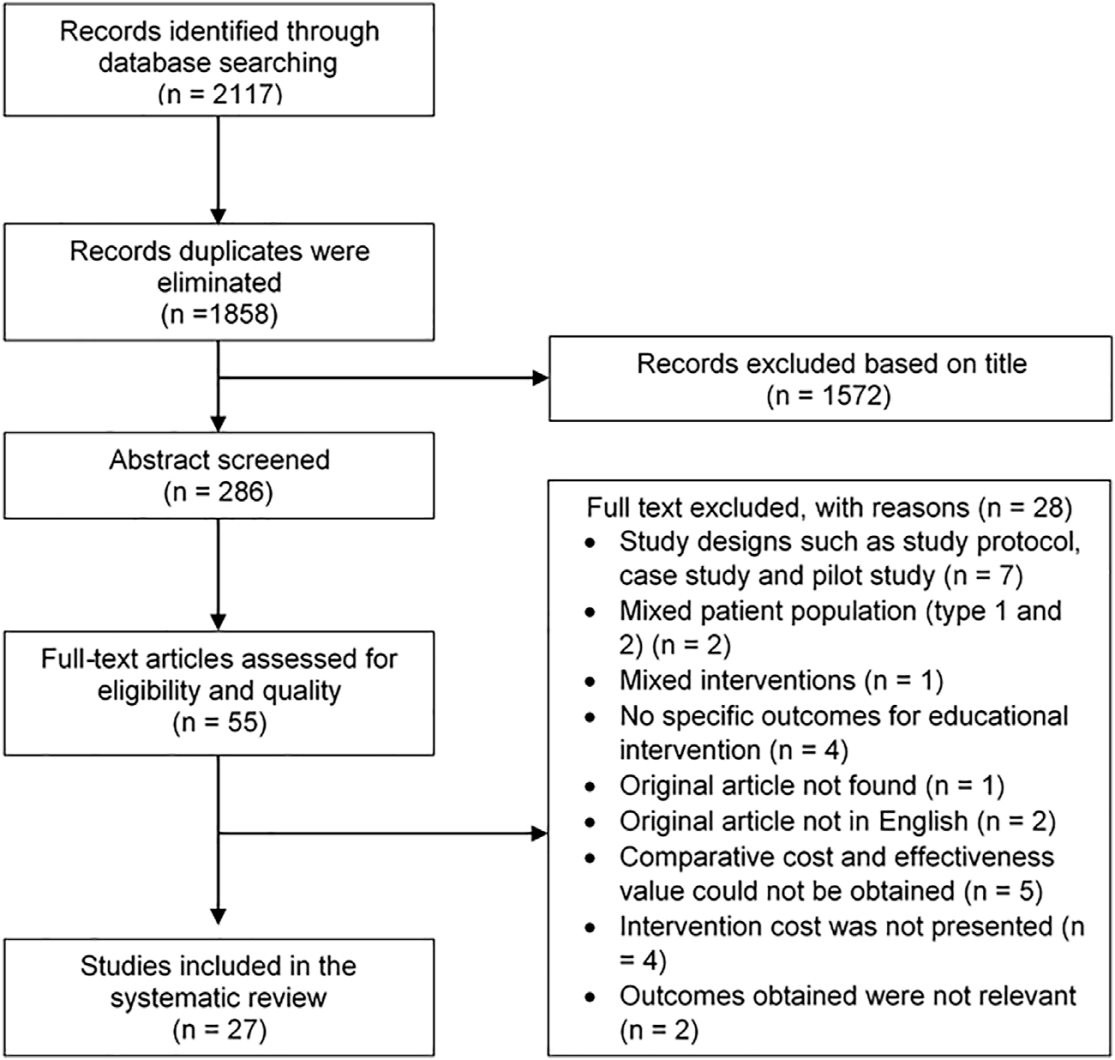
Flow diagram for selection of articles of a systematic review of the cost-effectiveness of different types of educational interventions in type II diabetes mellitus.

The study selection process was summarized in Figure 1. A total of 2,117 articles were generated from the database searches. 1858 records of duplicates were eliminated, and the title of the remaining records was screened. 1,572 records were excluded due to the irrelevance of the title, leaving 286 records to be screened based on their abstracts. This led to the further exclusion of 231 articles and a full-text assessment was performed on the remaining 55 articles. 28 articles were excluded for reasons as stated in Figure 1. As a result, 27 articles that were deemed to fulfill the inclusion criteria were included in the review.

### Study characteristics

The characteristics of the included study are tabulated in Table 1. Out of twenty-seven studies selected, thirteen were performed in European countries (Davies et al., 2001; Trento et al., 2002; Keers et al., 2005; Dijkstra et al., 2006; Simon et al., 2008; Molsted et al., 2012; Tao et al., 2015; Elliott et al., 2016; Odnoletkova et al., 2016; Elliott et al., 2017; Johansson et al., 2017; Oksman et al., 2017; Wu et al., 2018), eight studies were conducted in the United States (Banister et al., 2004; Handley et al., 2008; Brownson et al., 2009; Brennan et al., 2012; Schechter et al., 2012; Hendrie et al., 2014; Prezio et al., 2014; Schechter et al., 2016), while the remaining were in South Africa (n = 1) (Mash et al., 2015), India (n = 1) (Kesavadev et al., 2012), Australia (n = 2) (Gillett et al., 2010; Varney et al., 2016) and Hong Kong (n = 2) (Lian et al., 2017; Lian et al., 2019). The articles were published from 2001 to 2019, where most (N = 15, 56%) of the articles were published within the 10 years of the study.

**TABLE 1:** Study characteristics of a systematic review of the cost-effectiveness of different types of educational interventions in type II diabetes mellitus.

Author, year, country	^a^EE type; study design	Intervention	Comparator	Perspectives^a^; cost year	Type of cost	Sources of data	Time horizon	Main outcome measure
Banister et al. (2004), US	CEA (NS); Quasi-experimental design	“Diabetes Self-Management Training Program”, 4 h group education about self-monitoring, goal setting, and diabetes knowledge, followed by ≥ 1 individual consultation with a dietitian and support meeting once a month	Pre intervention	Provider (NS); (NA)	Program	Medical records	12 months	CE Ratio
Brennan et al. (2012), US	CBA (NS); Prospective Cohort	Integrated pharmacist-led educational intervention by distributing the learning materials *via* mail-order using brochures and short medication counseling sessions *via* call (automated/vice versa)	Pre-intervention	Healthcare; (NA)	Program	Medication adherence assessment, initiation rate of concomitant therapies	6 months	ROI
Brownson et al. (2009), US	CUA; Model-based EE (Markov)	Multifaceted national self-management and educational programs strategies involving home visits, skill-building education classes, counseling, and support group (depending on site of programs)	Pre-intervention	Health system; (NA)	Program, medical	Site visits, Patient surveys, Medical records	3–4 years	ICER (QALY)
Davies, et al. (2001), United Kingdom	CBA (NS); RCT	Individual structured patient education delivered by DSN to improve QOL and diabetic knowledge (assessed 1 week after discharge), combined with case-note feedback regarding practical management advice to staffs	UC	Healthcare (NS); 1997–8	Employer, Hospital admission	Four-point scale (Patient dependency), ADDQoL, Questionnaire	12 months	Cost reduction
Dijkstra et al. (2006), Netherlands	CEA; Model-based EE (Markov model)	Guideline-based educational intervention conducted for both professionals and patients group. For professional-directed group, opinion sharing about current guidelines were held, with the distribution of desktop card reminder containing guidelines statements as the aid. The patient-lefted group received a diabetes booklet and information leaflet containing information regarding diseases management	Patient- & Professional-directed group and UC	Societal (NS); (NA)	Program, medical	Empirical data from RCT in 13 hospitals	Lifetime	ICER (QALY)
Elliott et al. (2016), England (United Kingdom)	CBA (NS); RCT	Pharmacist-led consultation emphasizing medication-taking behaviors after 7–14 days of initial prescription presentation and follow-up after 14–21 days *via* telephone	UC	Payer (NS); (NA)	Program	Telephone interview, Postal questionnaire, Patient’s diaries	14 months	Cost reduction compared with NHS costs as a reference
Elliott et al. (2017), England (United Kingdom)	CEA: Model-based EE (Markov) based on RCT	Face-to-face/telephone-based pharmacist-led consultation about medication management and adherence after 7–14 days of initial prescription, with a follow-up after 14–21 days for 5 weeks	UC	Payer; 2015–16	Program	Patient diaries, Previous NHS data	Lifetime	ICER
Gillett et al. (2010), Australia	CUA; Model-based EE (Sheffield type 2 diabetes)	6h of DESMOND structured group education Program (1 full day/2-days of half-day session) by 2 professional healthcare educators	UC	Societal; (NA)	Program, medication	Data from an RCT	Lifetime	ICER (QALY)
Handley et al. (2008), US	CUA; RCT	Patient education *via* ATSM (automated interactive telephone technology) and 1-to-1 counseling by a nurse over 9 months	UC	Health system; (NA)	Program	12-Item Short-Form Health Survey	12 months	ICER
Hendrie et al. (2014), US	CEA (NS); RCT	1-to-1 sessions of DMEP delivered by pharmacist diabetes educators, together with follow-up visits at 1-,3- and 6 months + reminder calls. Each session can be up to 3 h. The health educators were trained on techniques about complex diabetes medication concepts and tailoring the education according to patient’s needs	UC	Health sector; 2011	Program	DPAQ Questionnaire, Diabetes diary	6 months	ICER
Johansson et al. (2017), Austria	CBA (NS); RCT	Self-management education Program using peer support and healthcare professionals for over 2 years consisting of 4 elements: peer supports recruitment + training + physical activity meetings + peer support meeting	UC	Societal (NS); (NA)	Program, Medication, Travel	Data from the previous RCT	24 months	Estimated yearly savings
Keers et al. (2005), Netherlands	CBA (NS); CCT (NS)	10 days of group educational sessions given by diabetes education team, individual support in 10 weeks and follow up visit on week 6, week 12 and 1-year post-intervention	Reference group	Limited societal; 2003	Program, Travel	Self-report, PAID Questionnaire	12 months	Cost reduction
Kesavadev et al. (2012), India	CBA (NS); Retrospective Cohort study	The educational intervention focused on glucometer training for 30–60 min, lifestyle (e.g., diet, exercise and other diabetic care) for 3 h s, followed up by DTMS consultation on phone/e-mail/website +1 half-day 4–5 h seminars every 2 months	Pre-intervention (The SMBG was not measured in usual care)	Provider (NS); (NA)	Program	Electronic health record system	6 months	Costs saving
Lian et al. (2017), Hong Kong	CEA (NS); CCT (NS)	A structured patient Empowerment Program (PEP) subjects consisted of 4 sessions (2.5 h for disease-specific sessions + 2 h for generic sessions)	Non-PEP	Societal; (NA)	Program, Travel	Structured questionnaire	5 years	ICER
Lian et al. (2019), Hong Kong	CEA (NS); Model-based EE (Patient simulation level model)	Patient Empowerment Program (PEP) subjects used to increase knowledge about DM and self-management skills, self-efficacy and lifestyle modification	Non-PEP	Societal; 2017	Program, Health care	Empirical data from PEP Program follow-up (SF-12v2 health survey)	Lifetime	ICER
Mash et al. (2015), South Africa	CEA; Model-based EE (Markov model)	4 sessions (60 min each) of group education using MI style emphasizing diabetic knowledge, lifestyle modification and medication management	UC	Societal; 2014	Program, Patient	Data from previous RCT	Lifetime	ICER
Molsted et al. (2011), Denmark	CBA (NS); CCT (NS)	Group education *via* 3 modules of DSME using empowerment strategy. Model 1 consists of 28 h (4 days), Model 2–14 h (2 days) and Model 3–7 h (1day), focusing on self-management improvement	Pre-intervention	Societal (NS); 2008	Program	Electronic database	12 months	Healthcare costs savings
Odnoletkova et al. (2016), Belgium	CEA; Model-based EE (Markov) based on RCT	5 sessions of telecoaching (COACH Program) with a timeframe of 30 min s each for 6 months on self-monitoring, lifestyle modification and intensification of medication treatment (after consultation with GP) after 1 week of training course	UC	Healthcare; (NA)	Program, Healthcare	Data from the previous RCT	40 years–lifetime	ICER
Oksman et al. (2017), Finland	CEA; RCT	Monthly motivational interviewing and health-coaching Program by telephone emphasizing self-management, self-efficacy, medication-taking behavior, lifestyle modifications and follow-up with specialists and appointments. Supplemented by booklets + TLS system	UC	Societal (NS); (NA)	Healthcare, Patient (home care + social care)	15D Questionnaire, Patient administration system (PAS)	12 months	ICER
Prezio et al. (2014), US	CEA (NS); Model-based EE (Archimedes)	7 sessions of 1-to-1 culturally tailored CoDE Program	UC	Health system; (NA)	Program	Data from previous RCT	5-,10- and 20-years	ICER
Schechter et al. (2012), US	CEA; RCT	10 sessions of behavioral counselling delivered by telephone every 4–6 weeks, focusing on medication adherence and lifestyle (eating and physical activity)	Print group (received brochure)	Provider; 2009	Program	Phone calls, record review	12 months	ICER
Schechter et al. (2016), US	CEA; RCT	4–8 behavioral counseling and self-management support given by health educators in addition to mailed printed materials and lifestyle incentives	Print group	Provider; 2013	Program	Questionnaire	12 months	ICER
Simon et al. (2008), United Kingdom	CUA; RCT	Self-monitoring of blood glucose level education, with the explanation on how to use a blood glucose meter and its application to diet, physical activity and drug adherence	UC	Healthcare; 2005–6	Program, medication, healthcare	Patients’ diaries, Nurses’ notes, Questionnaires, Medical records	12 months	QALY and healthcare costs
Tao et al. (2015), United Kingdom	CEA; RCT	Educational session focusing on lifestyle modifications (losing weight, physical activity, alcohol intake), medication taking behavior and self-monitoring given by GP + DESMOND given in Leicester	UC	Payer; 2009–10	Program, treatment	Self-report questionnaires	30 years	ICER
Trento et al. (2002), Italy	CBA (NS); RCT	4 systemic group education every 3 months about lifestyle (weight, food, physical activity, smoking) and medication use delivered annually (Year 1–2), changed to 7 sessions (Year 3–4) + special individual reinforcement follow-up (for those needed)	Individual consultations and education	Societal (NS); 1996–00	Program, healthcare	Questionnaire (GISED, CdR and DQoL/Mod)	4 years	ICER
Varney et al. (2016), Australia	CUA; Model-based EE (UKPDS) based on RCT	Diabetes management education counseling delivered *via* telephone coaching focusing on lifestyle modification, treatment compliance, goal setting and barriers to change for 6 months	UC	Health system; 2012–13	Health care	Data from DTCS	10 years	ICER (QALE)
Wu et al. (2018), US	CBA (NS); RCT	2 h education sessions *via* pharmacist-led medical-visits in a group 4–6 patients, (1st h - lifestyle modifications, 2nd h -medication management) once a week, conducted over 4 weeks and follow-up every 3 months	UC	Payer; 2012–3	Program, Medication, Healthcare	Medical records	13 months	Healthcare costs reduction

^Footnotes and Abbreviations: 15D, 15-dimensional; ADDQoL, Audit of Diabetes Dependent QoL; ATSM, Automated telephone self-management; CBA, Cost-Benefit Analysis; CCT, controlled clinical trial; CdR, condotte di riferimento; CEA, Cost-Effectiveness Analysis; CE, Cost-effectiveness; CoDE, community oriented diabetes education; CUA, Cost-Utility Analysis; DTCS, diabetes telephone coaching study; DSN, diabetes specialist nurse; DPAQ, diabetes patient assessment questionnaire; DQoL/Mod, Diabetes Quality of Life questionnaire (Modified); EE, economic evaluation; e.g, exemple gratia; GISED, education study group of the italian society for diabetes; GP, general practitioner; h, hour(s); ICER, Incremental cost-effectiveness ratio; min(s), minutes; MI, motivational interviewing; NA, Not available/applicable; NHS, national health service; NS, Not stated (Author’s judgement); PAID, problem areas in diabetes; PAS, patient administration system; PEP, patient empowerment program; QALE, Quality-Adjusted Life Expectancy; QALY, Quality-Adjusted Life Years; QOL, quality of life; RCT, randomized controlled trials; ROI, return on investment; SF-12v2, Short Form-12; items (Version 2); TLS, Traffic-light system; UC, usual care; United Kingdom, united kingdom; UKPDS, united kingdom prospective diabetes study; US, united states.^

a ^The author assumed the economic evaluation or the perspective of the study if it is not specifically stated in the article.^

Most of the studies compared the intervention group with the usual care practice, as well as the pre-intervention group (Banister et al., 2004; Brownson et al., 2009; Brennan et al., 2012; Kesavadev et al., 2012; Molsted et al., 2012) except for a few which compared them to their designated control group (Trento et al., 2002; Keers et al., 2005; Dijkstra et al., 2006; Schechter et al., 2012; Schechter et al., 2016; Lian et al., 2017; Lian et al., 2019).

The study design of economic evaluation incorporated in this study was model-based economic evaluation (n = 9) (Dijkstra et al., 2006; Brownson et al., 2009; Gillett et al., 2010; Prezio et al., 2014; Mash et al., 2015; Odnoletkova et al., 2016; Varney et al., 2016; Elliott et al., 2017; Lian et al., 2019), randomized controlled trial (n = 12) (Davies et al., 2001; Trento et al., 2002; Handley et al., 2008; Simon et al., 2008; Schechter et al., 2012; Hendrie et al., 2014; Tao et al., 2015; Elliott et al., 2016; Schechter et al., 2016; Johansson et al., 2017; Oksman et al., 2017; Wu et al., 2018) and prospective cohort studies (n = 2) (Brennan et al., 2012; Kesavadev et al., 2012). Another four studies were classified as quasi-experimental design (n = 1) (Banister et al., 2004) and controlled clinical trials (n = 3) (Keers et al., 2005; Molsted et al., 2012; Lian et al., 2017) as the authors did not explicitly clarify the nature of allocation of the participants in the study.

Eight studies were reported as cost-effectiveness analysis (CEA) (Dijkstra et al., 2006; Schechter et al., 2012; Mash et al., 2015; Tao et al., 2015; Odnoletkova et al., 2016; Schechter et al., 2016; Elliott et al., 2017; Oksman et al., 2017), while five studies were cost-utility analysis (CUA) with QALYs measured obtained as the results (Handley et al., 2008; Simon et al., 2008; Brownson et al., 2009; Gillett et al., 2010; Varney et al., 2016). Other studies (N = 14) did not mention the type of economic evaluation that was performed (Davies et al., 2001; Trento et al., 2002; Banister et al., 2004; Keers et al., 2005; Brennan et al., 2012; Kesavadev et al., 2012; Molsted et al., 2012; Hendrie et al., 2014; Prezio et al., 2014; Elliott et al., 2016; Johansson et al., 2017; Lian et al., 2017; Wu et al., 2018; Lian et al., 2019).

Overall, the type of costs identified in the studies includes the program costs, health care costs, travel costs, drug costs as well as out-of-pocket costs that needed to be paid by the people such as for home and social care. Most of the studies estimated the costs from a single site or multi-site evaluation, with the exclusion of three studies that generalizes the cost estimates from nationwide data (Gillett et al., 2010; Lian et al., 2017; Lian et al., 2019). Twelve studies were performed over 6 months up to 12 months (Davies et al., 2001; Banister et al., 2004; Keers et al., 2005; Handley et al., 2008; Simon et al., 2008; Brennan et al., 2012; Kesavadev et al., 2012; Molsted et al., 2012; Schechter et al., 2012; Hendrie et al., 2014; Schechter et al., 2016; Oksman et al., 2017), and 3 studies were conducted in the range of 13 months up to 24 months (Elliott et al., 2016; Johansson et al., 2017; Wu et al., 2018). Of these, the remaining twelve studies (Trento et al., 2002; Dijkstra et al., 2006; Brownson et al., 2009; Gillett et al., 2010; Prezio et al., 2014; Mash et al., 2015; Tao et al., 2015; Odnoletkova et al., 2016; Varney et al., 2016; Elliott et al., 2017; Lian et al., 2017; Lian et al., 2019) were extrapolated between 3 years to a lifetime horizon in estimating the long-term cost-effectiveness of the educational interventions.

### Evidence for the cost-effectiveness of educational interventions in improving health outcomes in T2DM

This section summarized the cost-effectiveness of each type of educational intervention. The types of educational interventions were divided into face-to-face education, structured education programs, telemedicine health education, others (peer education *via* discussion; self-management education, and support), and combination. To sum it up generally, out of the 27 studies included, only two educational interventions (Simon et al., 2008; Tao et al., 2015) were considered not cost-effective. The cost-effectiveness of one study (Davies et al., 2001) was not determined and the remaining economic evaluations have shown evidence of the cost-effectiveness of educational interventions delivered to T2DM people. Except for these three studies, the interventions were found to be cost-effective when compared to the comparator.

In general, seventeen studies have reported positive health outcomes linked to educational interventions. The changes in the HbA1c level were the most common health effect observed. The studies that utilized the HbA1c level as their main parameter demonstrated positive health benefits either a significant reduction of HbA1c level are achieved or the level remained controlled throughout the studies. Other significant positive outcomes include a reduction in the number of days of hyperglycaemic episodes, the development of complications, hospitalization rates, and mortality due to T2DM.

Furthermore, educational interventions were associated with cost reduction to obtain health benefits in nine studies (Davies et al., 2001; Banister et al., 2004; Keers et al., 2005; Brennan et al., 2012; Kesavadev et al., 2012; Molsted et al., 2012; Elliott et al., 2016; Johansson et al., 2017; Wu et al., 2018). Medication adherence assessments were only found to be performed in two studies (Brennan et al., 2012; Elliott et al., 2016). These details can be briefly viewed in Table 2.

**TABLE 2:** Effectiveness and economic outcomes of a systematic review of the cost-effectiveness of different types of educational interventions in type II diabetes mellitus.

Author, year, country	Medication adherence	Effectiveness (clinical measures/health effects)	Costs	Outcome(s) measures	Ratio
Banister et al. (2004), US	NA	Mean HbA1C level: 83 mmol/mol (9.7 ± 2.4% vs. 66 mmol/mol (8.2 ± 2.0%), (*p* < 0.001) Medication positive outcomes: 61%	Total Program cost: US$35,436 Costs/patient: US$279 Emergency departments visit: US$450	Saved 38% < 1 emergency admission	CE Ratio: US$185/A1C point
Brennan et al. (2012), US	% change (during/post-program) Full sample: 2.1 vs. 1.0 Retail group: 3.9 vs. 1.2 Pharmacy benefit management group: 1.7 vs. 1.0	NA	Costs/person: US$1.00 Program cost: ∼ US$200,000/63,000 beneficiaries	> US$600,000 saved/63000 beneficiaries	ROI = 3:1
Brownson et al. (2009), US	NA	NA	Total cost: US$61 234 vs. US$49 474	LY (undiscounted): 21.8849 vs. 21.3434 QALY: 14.6541 vs. 14.3569	ICER = US$39 563
Davies et al. (2001), United Kingdom	NA	Median LOS: 8 vs. 11 days (*p* < 0.01) Readmission rate: Equal for both groups (25%) Readmission time: 278 vs. 283 days (*p* = 0.80)	Total cost: £30,064 Cost/patient: £38.94	Mean cost of £436 reduced	NA
Dijkstra et al. (2006), Netherlands	NA	Post HbA1c level: 0.3% (Patient-centred)-0.1% (Professional-directed) +0.2% (Control); *p* < 0.001	Costs/patient (Professional-directed): €2	LE: 0.34 vs. 0.63	Incremental cost/QALY: €32,218 (Professional-directed) vs. €16 353 (Patient-centred)
			Costs/patient (Patient-centred): €3	QALY: 0.29 vs. 0.59	
			Lifetime costs: €9389 vs. €9620		
Elliott et al. (2016), England (United Kingdom)	Increased adherence in NMS group with an odds ratio of 1.67	Health beliefs: No changes	Mean total NHS cost (UC vs. NMS): £261 vs. £ 231	NA	NA
		Health status: No changes	£21 NHS cost averted for NMS intervention/patient		
Elliott et al. (2017), England (United Kingdom)	NA	NA	Medication cost: £8.05	Overall cost reduction: £139 to £144	ICER: £293
			Mean Program costs: £15,285.7 vs. 15,279.8	QALY: 9.55 vs. 9.53	
				Incremental costs: £5.9	
Gillett et al. (2010), Australia	NA	Difference of biomarkers level at 12 months: HbA1c level: 0.06%	Total cost (program + drug use): £219 (trial costs) and £92 (real world costs) vs. £244	QALY at 12 months: 0.7600 vs. 0.7530	NA
		Total cholesterol: −0.044 mmol/L		Combined lifetime QALY: 10.0026 vs. 9.9634 (difference: 0.0392)	
		HDL-cholesterol: 0.015 mmol/L	Estimated lifetime incremental costs: £209 (trial costs) and £82 real-world costs)	Incremental cost/QALY	
		Systolic BP: 0.984 mmHg		£5387 (trial costs) and £2092 (real world costs)	
Handley et al. (2008), US	NA	NA	Total cost annually: US$782	QALY: 0.012 (Intervention)	US$65,167/QALY (Start-up + Ongoing cost)
			Cost for 10% increase of patient achieving standard exercise guidelines: US$558 (All costs considered) US$277 (Ongoing cost considered)		US$32,333/QALY (Ongoing cost only)
Hendrie et al. (2014), US	NA	Average no. of days of hyperglycemic episodes per month: 3.40 vs. 3.95 (baseline) 1.07 vs. 2.88 (post-intervention)	Total costs: AU$27,591	Total days of hyperglycemic and hypoglycemic episodes avoided for 6 months: 11.16 days	ICER = AU$43/days of glycemic episodes avoided
		No. of days of hypoglycemic episodes per month: 0.97 vs. 0.79 (baseline) 0.42 vs. 0.84 (post-intervention)	Total costs/patient: AU$394		
Johansson et al. (2017), Austria	NA	Mean prescribed drugs: 892.9 vs. 1,003.5	Total costs: €49 725.90	The estimated cost saved for hospital admission: €4241/patient	NA
		Mean all-cause hospital admissions: 10.2 vs. 12.1 days	Costs/patient: €210.70	The estimated yearly cost saved (intervention cost and drugs cost considered): €1,660.60/patient	
		Mean length of stay: 65.3 vs. 105.4 days	Mean costs of prescribed drugs: 18,406.1 vs. 16,206.8		
Keers et al. (2005), Netherlands	NA	HbA1c level: 69 mmol/mol (8.5 ± 1.3) (baseline) 65 mmol/mol (8.1 ± 1.2) (1 year after) 64 mmol/mol (8.0 ± 1.2) (reference group)	Total program + travel cost: €1,327	Mean reduction of costs post-program due to reduction of HbA1c: €2,144 vs. €509	NA
		Occurrence of hypoglycemia: 9.3 ± 8.1 (baseline) 5.7 ± 5.9 (1 year after) 5.6 ± 6.8 (reference group)		Mean reduction of costs post-program in achieving PAID scores reduction: €2,535 vs. €408	
		Occurrence of ≥1 severe hypoglycemia: 18% (baseline) 12% (1 year after) 14% (reference group)		Overall costs reduction: €2025 vs. €499 (*p =* 0.13)	
		PAID scores (total): 38 ± 22 (baseline) 22 ± 15 (1 year after) 25 ± 18 (reference group)			
Kesavadev, et al. (2012), India	NA	HbA1c level: 69 mmol/mol (8.5 ± 1.4) (baseline) 45 mmol/mol (6.3 ± 0.6) (post 6-months)	Total Program Cost: US$38.04/6 months	Saved US$9.66 (INR 456.92)/patient/month for patients requiring intensive treatment	NA
		FBS: 174 (baseline) vs. 107	Total cost for each patient: US$6.34 (∼INR 300)/patient/month		
		LDL: 126 (baseline) vs. 82	Reporting values cost: US$0.07 (INR 3.31)/patient/month		
		Triglycerides: 137 (baseline) vs. 102	Telemedicine services cost: US$3.25/patient/month		
		Total cholesterol: 194 (baseline) vs. 138	Cost to attend physical visit: US$5 to US$15		
Lian et al. (2017), Hong Kong	NA	Frequency of all-cause mortality: 2.9 vs. 4.6%, *p* < 0.001	Total Program cost: US$191 - US$297	NA	ICER of all-cause mortality: US$14,465
		Frequency of DM-related complications mortality: 9.5 vs. 10.8%, *p* = 0.001	Average Program Cost: US$247/patient		ICER of DM-related complications mortality: US$19,617
		Frequency of CVD-related mortality: 6.8 vs. 7.6%, *p* = 0.018	Costs to avoid CVD-related cost: US$68,192		ICER of CVD-related mortality: US$30,796
					ICER to avoid stroke death: US$42,747
					ICER to avoid HF: US$58,450
Lian et al. (2019), Hong Kong	NA	NA	Annual Program cost: US$276/patient	Incremental cost: US$197 for 0.06 QALY	ICER: US$3,290/QALY achieved
			Lifetime cost: US$30,621 (PEP group) US$30,423 (Non-PEP group)		
Mash et al. (2015), South Africa	NA	NA	Salary costs: US$2082	Cost/patient/year: US$22 to avoid mortality	ICER: Annual with persistent benefit = US$1862/QALY
			Total training costs: US$6958		1-year cost with persistent/1 year/3 years benefits = Dominant/QALY
			Total educational material cost: US$949		
			Operational costs: US$110		
			Patient costs: US$8132		
Molsted et al. (2011), Denmark	NA	HbA1c level: 57 mmol/mol (7.34 ± 1.34) (Module 1) vs. 53 mmol/mol (7.00 ± 1.15) (Module 2) vs. 52 mmol/mol (6.88 ± 1.09) (Module 3)	Cost/patient: DKK 3640 (€489) Potential cost-saving/patient/year for physical visits: DKK 226 (€30)	Total cost-saving/patient to avoid hospitalizations: DKK 423 (€56)	NA
		FBS: 8.7 ± 2.6 (Module 1) vs. 7.8 ± 2.3 (Module 3)			
		Systolic BP: 138.2 ± 15.3 (Module 1) vs. 137.7 ± 15.8 (Module 2) vs137.1 ± 15.4 (Module 3)			
		Diastolic BP: 81.6 ± 9.3 (Module 1) vs. 80.0 ± 9.2 (Module 2) 79.4 ± 9.0 (Module 3)			
		Total cholesterol: 4.88 ± 1.09 (Module 1) vs. 4.46 ± 0.91 (Module 3)			
		Triglyceride level: 1.92 ± 1.39 (Module 1) vs. 1.59 ± 1.09 (Module 3)			
		LDL level: 2.69 ± 0.97 (Module 1) vs. 2.29 ± 0.76 (Module 3)			
		HDL level: 1.36 ± 0.43 (Module 1) vs. 1.44 ± 0.42 (Module 3)			
Odnoletkova et al. (2016), Belgium	NA	NA	Intervention cost: €300.3	QALY gained: 0.21 (All patients) 0.56 (Subgroup)	Mean ICER: €5,569/QALY (All patients) €4,615/QALY (Subgroup)
			Within trial cost: €5,516		
			Incremental long-term cost: €1,147 (All patients) €2,565 (Subgroup)		
Oksman et al. (2017), Finland	NA	NA	Overall costs (T2DM vs. CAD vs. CHF): 2,256 vs. 1824	Overall QoL (T2DM vs. CAD vs. CHF): 0.011 vs. 0.002	ICER for T2DM: €20000/QALY
			T2DM costs: 948 vs. 1788	QoL of T2DM: 0.008 vs. 0.000	Overall ICER: €48000/QALY
Prezio et al. (2014), US	NA	HbA1c: 60 mmol/mol (7.61%) vs. 70 mmol/mol (8.55%)	Opportunity cost/patient (1st year): US$435	Overall LY gained: 354.00	ICER/QALY-5years: $100,195
		Foot ulcer: 55.69 vs. 64.34	Opportunity cost/patient (the following years): US$316	Overall undiscounted QALY gained: 841.99	ICER/QALY-10years: US$38,726
		Foot amp: 12.85 vs. 16.02	Estimated costs (20 years): US$4,958 ($0.68 per day)–3% discount rate	Overall QALY (discounted 3%) gained: 561.33	ICER/QALY-20years: US$355
Schechter et al. (2012), US	NA	HbA1c % point reduction: 0.36	Intervention cost/person: US$176.61	NA	ICER: For 0.36% reduction: US$490.58
			Educational materials: US$4.00		In attaining <7% HbA1c level: US$2,617.35
Schechter et al. (2016), US	NA	Mean difference of HbA1c level: 0.43 (95% CI 0.09–0.74)	Total Program cost: US$419.52	NA	CE ratio: US$2,109.25
			Direct cost/participant: US$187.61		
Simon, et al. (2008), United Kingdom	NA	HbA1c level (less intensive vs. control): 0.14% (*p* = 0.12)	Average costs of intervention: £89 (UC) vs. £181 (less intensive) vs. £173 (more intensive)	QALY gained: 0 (UC) vs. -0.008 (less intensive) vs. -0.035 (more intensive)	NA
		HbA1c level (more intensive vs. control): 0.17%	Overall costs difference for intervention/patient: £92 (less intensive vs. control), £84 (more intensive vs. control)		
		Glucometer use persistence: 67% (less intensive) vs. 52% (more intensive)			
Tao, et al. (2015), United Kingdom	NA	NA	Total Program cost: £502,974		ICER: 5years: US$100,195
			Costs/patient: £981		ICER/QALY-10years: US$38,726
					ICER/QALY-20years: US$355
Trento et al. (2002), Italy	NA	HbA1c level: 53 mmol/mol (7.0 ± 1.1) vs. 70 mmol/mol (8.6 ± 2.1)	Staffs’ and materials cost: US$108.87 vs. US$82.50	Costs/patient: US$756.54 for 196 min spent vs. US$665.77 for 150 min s spent for educational sessions	US$2.12/QoL point score
		BMI: 28.7 ± 4.0 vs. 27.6 ± 4.7	Drug cost/patient: US$0.26 vs. US$0.23 (baseline) US$0.36 and US$0.44 (4th year)		
		HDL-cholesterol: 1.42 ± 0.31 vs. 1.37 ± 0.28	Total drug costs/patient: US$ 488.57 vs. US$488.02		
		Diastolic BP: 88 ± 7 vs. 86 ± 9			
Varney et al. (2016), Australia	NA	HbA1c: −0.8% (95% CI)	Cost of intervention: AU$8581 vs. 0	Costs: AU$3327 saved to gain 0.2 QALY	Stroke: AU$4365/QALY
		The 10-years risk of complications: 32 vs. 38%	Cost of complications: AU$51,210 vs. AU$63,117		Cost of complications: AU$7425/QALY
		The 10-years risk of death: 32 vs. 30	Total discounted cost: AU$59,790 vs. AU$63,117		Cost of no complications: AU$45,605/QALY
		Life expectancy: 8.1 vs. 7.7 years			
		Total QALE: 4.9 vs. 4.7 years			
Wu et al. (2018), US	NA	UKPDS risk scores: 0.02 ± 0.09 vs. -0.04 ± 0.09, *p* < 0.05	Cost of group visits/patient: $370 ± 192	Healthcare cost reduction/patient: -$1,575 ± 30,774 (-5.9%) vs. +$2,360 ± 23,708 (+13.2%) adj. *p* = 0.05	NA
		HbA1c: 0.27 ± 1.25% vs. -0.14 ± 1.23%, *p* = 0.30	Health care service costs/patient: +$4656 (+21.1%)4+$2,645 (+17.4%)		
		Systolic BP: 6.9 ± 19.7 vs. -8.9 ± 17.4 mmHg, *p* = 0.12	Outpatient cost/patient: +$1,629 vs. +$1943; adj. *p* = 0.04 (during study) -$795 vs. +$501; adj. *p* < 0.01 (post 13 months)		
		LDL-cholesterol: 5.4 ± 30.1 vs. -14.2 ± 30.0 mg/dl, *p* = 0.12	Medication cost/patient: +$1,213 vs. +$318; adj. *p* = 0.03 (during study) -$331 vs. +$655 adj. *p* = 0.29 (post 13 months)		
		QoL (SF36v): 117 vs. 132 (baseline) 99 vs. 119 (post 13 months)			

Abbreviations: €, Euro; %, Percentage; ±, Plus-minus; £, Pound; AU$, Australian dollar; amp., Amputation; BP, Blood pressure; CAD, Coronary Artery Disease; CE, Cost-effectiveness; CHF, Congestive Heart Failure; DKK, Denmark Danish Krone; DM, Diabetes mellitus; FBS, Fasting blood sugar; HbA1c, Haemoglobin A1c; HDL, High-density lipoprotein; ICER, Incremental cost-effectiveness ratio; LOS, Length of stay, LY, Life-Years; NA, Not available; NHS, National Health Service; NMS, National Medicine Service; no., number; QALY, Quality-Adjusted Life Years; QoL, Quality of Life ROI, Return on Investment; UKPDS, the United Kingdom Prospective Diabetes Study; US$, United States Dollar

### Face-to-face education

There are a total of four studies that conducted group-based education, while the remaining three interventions performed the education session individually. All studies were reported to be cost-effective and cost-saving (Banister et al., 2004; Keers et al., 2005; Wu et al., 2018) along with their positive clinical effects. The clinical findings showed a reduction in HbA1c levels (Trento et al., 2002; Banister et al., 2004; Keers et al., 2005; Prezio et al., 2014), hyperglycaemic and hypoglycaemic episodes (Hendrie et al., 2014), diabetes-related stress scores (Keers et al., 2005), and other biomarkers level (Trento et al., 2002). However, Mash et al. (2015) did not report the clinical effects resulting from the intervention given, and no clinical benefits were gained from one study (Wu et al., 2018). Medication adherence assessments were not conducted in any of the studies.

### Structured education programs

A total of six studies assessed the structured education programs delivered in the management of T2DM people with the majority (n = 5) reporting favorable cost-effectiveness outcomes. A study reported an inconclusive cost-effectiveness result (Davies et al., 2001). The study by Moldsted et al. (2012) presented a cost reduction associated with the implementation of the program (Molsted et al., 2012). The health advantages obtained from this component of intervention include a shorter readmission rate (Davies et al., 2001), lower HbA1c level (Dijkstra et al., 2006), other biomarkers levels (Gillett et al., 2010), mortality (Lian et al., 2017; Lian et al., 2019), and fewer physical visits (Molsted et al., 2012).

### Telemedicine health education

A total of six studies incorporated in the review discussed the implementation of the telemedicine approach to provide health education. Only one study reported a higher medication adherence (Brennan et al., 2012). Besides that, telemedicine health education was considered cost-effective in four studies and cost reduction was observed in two studies (Brennan et al., 2012; Kesavadev et al., 2012). T2DM people receiving this intervention were presented with reduced blood glucose and other biomarker levels (Kesavadev et al., 2012; Schechter et al., 2012; Varney et al., 2016); a lower risk of complications (Varney et al., 2016), and a higher quality of life (Oksman et al., 2017). Two studies did not report any clinical changes (Brennan et al., 2012; Oksman et al., 2017).

### Combined types of educational interventions

The remaining six studies utilized the combinations of educational interventions mentioned above for the management of people with T2DM. The majority of the studies demonstrated cost-effectiveness and cost reduction except for one study that concluded that the intervention is not cost-effective (Tao et al., 2015). Only one study recorded a reduction in HbA1c level (Schechter et al., 2016) while the remainder of the papers did not provide clinical effectiveness estimates. Improvements in medication adherence were also observed in a study by Elliott et al. (2016).

### Others

Only one study assessed peer discussions related to the management and treatment of T2DM as a type of health educational strategy (Johansson et al., 2017). It was found to be cost-saving and associated with positive health outcomes including hospital admissions. Another study found that integrating self-management education and people support had negative health outcomes and was not cost-effective for T2DM people (Simon et al., 2008).

### Quality assessment

The results of the quality assessment were presented in Table 3. About half of the studies (n = 13) included in the review were classified as high quality (Dijkstra et al., 2006; Simon et al., 2008; Brownson et al., 2009; Gillett et al., 2010; Hendrie et al., 2014; Prezio et al., 2014; Tao et al., 2015; Odnoletkova et al., 2016; Schechter et al., 2016; Varney et al., 2016; Elliott et al., 2017; Lian et al., 2017; Lian et al., 2019), while the other half (n = 13) demonstrated a modest-quality (Davies et al., 2001; Trento et al., 2002; Keers et al., 2005; Handley et al., 2008; Brennan et al., 2012; Kesavadev et al., 2012; Molsted et al., 2012; Mash et al., 2015; Elliott et al., 2016; Schechter et al., 2016; Johansson et al., 2017; Oksman et al., 2017; Wu et al., 2018). Only one study was subjected to poor quality (Banister et al., 2004).

**TABLE 3:** Quality assessment of a systematic review of the cost-effectiveness of different types of educational interventions in type II diabetes mellitus.

Author, year, country	1	2	3	4	5	6	7	8	9	10	Score^a^
Banister et al. (2004), US	NO	YES	YES	YES	NO	NO	NA	NO	NO	NO	3
Brennan et al. (2012), US	YES	YES	YES	YES	NO	NO	NA	NO	NO	NO	4
Brownson et al. (2009), US	YES	NO	YES	YES	YES	YES	YES	YES	YES	YES	9
Davies et al. (2001), United Kingdom	NO	YES	YES	YES	YES	YES	NA	NO	YES	NO	6
Dijkstra et al. (2006), Netherlands	NO	YES	YES	YES	YES	YES	YES	YES	YES	YES	9
Elliott et al. (2016), England (United Kingdom)	NO	YES	YES	YES	NO	NO	NO	NO	YES	YES	5
Elliott et al. (2017), England (United Kingdom)	YES	YES	NO	YES	YES	YES	YES	YES	YES	YES	9
Gillett et al. (2010),Australia	YES	NO	YES	YES	YES	YES	YES	YES	YES	YES	9
Handley et al. (2008), US	YES	NO	NO	YES	YES	YES	NA	NO	YES	YES	6
Hendrie et al. (2014), US	YES	YES	YES	YES	YES	NO	YES	YES	YES	YES	9
Johansson et al. (2017), Austria	NO	YES	YES	YES	NO	YES	NO	NO	NO	YES	5
Keers et al. (2005), Netherlands	YES	YES	YES	YES	NO	NO	NA	NO	NO	YES	5
Kesavadev et al. (2012), India	NO	NO	YES	YES	YES	NO	NA	NO	NO	YES	4
Lian et al. (2017), Hong Kong	YES	YES	YES	YES	YES	YES	NO	YES	YES	YES	9
Lian et al. (2019), Hong Kong	YES	YES	NO	YES	YES	YES	YES	YES	YES	YES	9
Mash et al. (2015), South Africa	YES	YES	NO	YES	YES	YES	NO	YES	NO	YES	7
Molsted et al. (2012), Denmark	NO	YES	YES	YES	YES	YES	NA	NO	NO	YES	6
Odnoletkova et al. (2016), Belgium	YES	YES	NO	YES	YES	YES	YES	YES	YES	YES	9
Oksman et al. (2017), Finland	NO	YES	NO	YES	YES	NO	NA	YES	YES	YES	6
Prezio et al. (2014), US	YES	YES	YES	YES	YES	YES	YES	YES	YES	YES	10
Schechter et al. (2012), US	YES	YES	NO	YES	NO	NO	NA	YES	YES	YES	6
Schechter et al. (2016), US	YES	YES	YES	YES	NO	YES	NA	YES	YES	YES	8
Simon et al. (2008), United Kingdom	YES	YES	NO	YES	YES	YES	NA	YES	YES	YES	8
Tao et al. (2015), United Kingdom	YES	YES	NO	YES	YES	YES	YES	YES	YES	YES	9
Trento et al. (2002), Italy	NO	YES	YES	YES	YES	YES	NO	YES	NO	YES	7
Varney et al. (2016), Australia	YES	YES	YES	YES	YES	YES	YES	YES	YES	YES	10
Wu et al. (2018), US	YES	YES	YES	YES	NO	NO	NO	NO	YES	NO	5
Total	18	23	18	27	19	18	10	17	19	23	—

Footnotes and Abbreviations: YES, presented; NO, not presented; NA, not applicable

a The sum of scores for meeting the specified criteria.

Twenty-three studies (excluding (Handley et al., 2008; Brownson et al., 2009; Gillett et al., 2010; Kesavadev et al., 2012)) successfully provided a detailed explanation regarding the comparator in the studies. The relevant costs and effects of the interventions were sufficiently established in all studies (n = 27). Almost 70% of the studies (excluding (Banister et al., 2004; Keers et al., 2005; Brennan et al., 2012; Schechter et al., 2012; Elliott et al., 2016; Schechter et al., 2016; Johansson et al., 2017; Wu et al., 2018)) expressed the outcomes according to their appropriate physical units, while another 67% (excluding (Banister et al., 2004; Keers et al., 2005; Brennan et al., 2012; Kesavadev et al., 2012; Schechter et al., 2012; Hendrie et al., 2014; Elliott et al., 2016; Oksman et al., 2017; Wu et al., 2018)) were able to report the credible value of its overall costs and effects. Only six studies (6/16, 37.5%) were unable to report the use of discounting approach for studies longer than 12 months in duration that required timing adjustments (Trento et al., 2002; Mash et al., 2015; Elliott et al., 2016; Johansson et al., 2017; Lian et al., 2017; Wu et al., 2018). Meanwhile, the robustness of the outcomes of the study cannot be confirmed in eight studies as sensitivity analyses were not conducted (Trento et al., 2002; Banister et al., 2004; Keers et al., 2005; Brennan et al., 2012; Kesavadev et al., 2012; Molsted et al., 2012; Mash et al., 2015; Johansson et al., 2017). Twenty-three studies (excluding (Davies et al., 2001; Banister et al., 2004; Brennan et al., 2012; Wu et al., 2018)) took into consideration the transferability of the outcomes concerning the use of interventions in the real world. The explicit incremental cost-effectiveness ratio (ICER) value was presented in seventeen studies [excluding (Davies et al., 2001; Banister et al., 2004; Keers et al., 2005; Handley et al., 2008; Brennan et al., 2012; Kesavadev et al., 2012; Molsted et al., 2012; Elliott et al., 2016; Johansson et al., 2017; Wu et al., 2018)].

Eighteen studies described the view of economic perspectives such as health care and health system (Handley et al., 2008; Simon et al., 2008; Brownson et al., 2009; Brennan et al., 2012; Hendrie et al., 2014; Prezio et al., 2014; Odnoletkova et al., 2016; Varney et al., 2016), societal (Keers et al., 2005; Gillett et al., 2010; Mash et al., 2015; Lian et al., 2017; Lian et al., 2019), provider (Schechter et al., 2012; Schechter et al., 2016) and payer (Tao et al., 2015; Elliott et al., 2017; Wu et al., 2018).

Furthermore, fourteen studies presented the year where the costs were indexed, ranging from 1997 up to 2017 (Davies et al., 2001; Trento et al., 2002; Keers et al., 2005; Simon et al., 2008; Molsted et al., 2012; Schechter et al., 2012; Hendrie et al., 2014; Mash et al., 2015; Tao et al., 2015; Schechter et al., 2016; Varney et al., 2016; Elliott et al., 2017; Wu et al., 2018; Lian et al., 2019). The study by Banister et al. (2004) was still included because they adequately documented the comparative outcomes between the costs and health benefits despite the poor methodology conduct (Banister et al., 2004).

## Discussion

Successful and cost-effective strategies to prevent or delay the onset and progression of T2DM are necessary to relieve the clinical and economic burden borne by the health care system and the people. Few studies reported promising outcomes over the implementation of health education for T2DM people. This systematic review gathers available evidence in providing updated information on the cost-effectiveness of types of educational interventions in improving peoples’ medication adherence and treatment compliance. The inclusion of various types of educational interventions is regarded as one of the strengths in this review for providing a more holistic view of its cost-effectiveness values. To summarize, 24 studies have shown to be cost-effective while the remaining three studies yielded a different conclusion. A study classified as a combined type of approach (Tao et al., 2015) and other types utilizing self-management education and people support (Simon et al., 2008) were determined to be not cost-effective. Davies et al. (2001) found the cost-effectiveness of structured programs to be inconclusive, owing to insufficient economic assessment (Davies et al., 2001). The majority of the studies were classified as having moderate-to-high quality studies.

Overall, the most cost-effective type of educational intervention cannot be determined due to the differences in the adopted perspective and methods in those studies. The differences in the reported outcomes of the studies cannot be compared directly across the categories of interventions because each study employed a different approach to economic evaluations, each with a specific purpose, comprising of various descriptions of perspectives and intended outcomes. The preferences, values, and criteria of the stakeholders requiring health economic assessment data are responsible for the selection of types of economic evaluation. The types of economic evaluations will address a wide range of objectives such as productive efficiency, allocative efficiency, social welfare, and policy depending on the description of the value of health from the perspective of the specified stakeholders. In this review, about half of the studies were in the form of either CEA or CUA, which mainly focuses on the productive efficiency of the interventions and some issues of allocative efficiency. This will also have an impact on the costing methods such as identification, measurement, precision, and valuation of the cost estimates as well as the weighting of various aspects of health outcomes. Although the societal perspective is recommended to be used for economic evaluation because it encompasses a wider range of viewpoints, only Keers et al. (2005) applied this perspective. Still, Keers et al. (2005) did not consider all costs such as intangible costs that are necessary to represent the social value. Furthermore, the level of precision of estimates varies widely for the program costs. For example, the program costs in a study by Banister et al. (2004) include the costs of educators’ salaries, glucometer kits, testing strips, and room rentals while Molsted et al. (2012) cover the costs for the educators’ salary, education materials, and maintenance. This, in turn, leads to different results and recommendations depending on the perspective attributed to the analysis. A larger number of studies should be included in the next review to allow for the comparison of studies using similar economic evaluation approaches to produce more homogeneous results. Furthermore, researchers are recommended to use standard guidelines to conduct an economic evaluation to improve the reliability of the outcomes. Furthermore, improving transparency and reporting in original studies and developments of frameworks and tools will further aid in the assessment of transferability issues in health economic evaluations (Kim et al., 2019; García-Mochón et al., 2021; Weise et al., 2022). Additionally, multi-national economic evaluations, international cost catalogues, and an open-source platform are prospective approaches to improving the transferability (Kim et al., 2019).

The timing issue is the main thing to be addressed in the study design, to have a good approximation and relevance of specified costs and health outcomes with T2DM disease progression. This is especially important and relevant in the case of T2DM because their financial and clinical implications become apparent only after years of interventions. In contrast to clinical trials, model-based economic evaluation is generally useful in providing evidence of long-term cost-effectiveness (Brownson et al., 2009). Long-term effects and costs are excluded from a trial design (Elliott et al., 2017), and performing a trial for long-term assessment could lead to a very costly and impractical intervention (Brownson et al., 2009). The employed time horizon also affected the value of health and economic outcomes, whereby in this review, the shortest time horizon recorded was 6 months (Kesavadev et al., 2012; Hendrie et al., 2014). The relatively short time horizon could not adequately evaluate the full potential of the cost-effectiveness of the diabetes management education program (Hendrie et al., 2014). For example, the emergence of T2DM complications and mortality better represent the overall and long-term effectiveness of interventions, and these outcomes could only be captured in a long-term study design. A longer time horizon may yield the opposite outcomes, where costs associated with reductions in disease progression to improve quality of life can be captured, proving the robustness of the cost-effectiveness in a pragmatic long-term duration (Brownson et al., 2009). The positive cost-effectiveness of all model-based studies in this review, with some even projecting to the lifetime horizon, may represent that the cost-effectiveness of educational intervention can be sustained over time. However, the cost-effectiveness results from the model-based design should be interpreted with caution considering a few assumptions and limitations made for the projection of the outcomes.

To date, the closest review to our study would be the systematic review of the cost-effectiveness of people education models by Loveman et al. (2003) in the management of DM. This review includes both type I and type II diabetes mellitus, and the author concluded that education as part of intensified treatment intervention in type I people could result in improvement in metabolic control and decrease the risk of developing diabetes-related complications. In contrast, inconsistent results were obtained for T2DM mainly due to a diversity of educational programs. In comparison to our study, the studies included were outdated as most of them were published in the 1990s, ranging from 1985 up to 2002. The authors also concluded that the quality of reporting and methodology of the studies was generally poor by today’s standards. Even, the most recent systematic review identified includes articles up to the publication date of 2014 and concludes that there are growing numbers of studies during the study period (Odnoletkova et al., 2014). Our review further updated the evidence where all the articles were published in the 2000s, which most concentrated from the year 2010. With further improvement and acceptance of the guidelines to conduct economic evaluation, we found that most of the articles published in past years adequately satisfy the quality standards of methodological conduct. Apart from updating the current evidence and quality standards of published articles, our review comprises all study designs including trials, cohort, and model-based studies, to provide a comprehensive overview of the shreds of evidence on educational interventions.

Another issue worth mentioning is regarded to publication bias, in the context of the study’s settings. The majority of the studies were concentrated in developed countries, where resources and skills for implementing educational interventions are well established. The need to provide training and development of materials for the program in low- and middle-income countries may require extra costs and resources. This may have an impact on the cost-effectiveness values of educational programs in this setting. Furthermore, the hypothetical study design of some model-based economic evaluations may limit the transferability of the findings in a real-world setting.

Nonetheless, there were some limitations to this review. The exclusion of non-English articles due to language barriers and Grey literature may reduce the number of articles retrieved, eventually affecting the quantity and comprehensiveness of evidence.

This systematic review sheds light on the cost-effectiveness of educational interventions in achieving optimal and planned diabetes care in managing T2DM. An appropriate and adequate knowledge and skills to manage T2DM and its complications are the necessary elements to achieve the effects of educational intervention, regardless of the type of educational intervention. Enhancement of awareness, knowledge, attitude and self-care are the main elements of educational interventions in helping people to manage diabetes and its complications as well as the need to adopt a positive lifestyle. Although the characteristics and magnitude of implementation of educational interventions are different, most of the interventions are considered cost-effective to improve the health benefits and quality of life of the people. The identified educational interventions would result in positive health benefits, lowering the risk of complications and improving the quality of life eventually leading to a significant reduction in the burden cost of this disease on the people and the health care system. Good quality and preferably long-term health economic studies utilizing societal perspectives are still needed. High-quality evidence will help in guiding and improving the healthcare decision-making process and allow the proper allocation of healthcare resources in the effort of maximizing the health benefits. More research that correlates the economic evaluation and aspects of educational interventions is needed to allow a more comprehensive implication of these interventions on the people’s awareness, knowledge, attitude, and self-enhancement toward T2DM care.

## Conclusion

All types of educational interventions are highly likely to be cost-effective. The quality of economic evaluations is moderate but the most cost-effective types of educational interventions could not be determined due to variations in the reporting and methodological conduct of the study. A high-quality approach, preferably utilizing the societal perspective over a long period, should be standardized to conduct economic evaluations for educational interventions in T2DM.

## Data Availability

The original contributions presented in the study are included in the article/supplementary material, further inquiries can be directed to the corresponding author.
